# Potential role of periodontal infection in respiratory diseases-a review

**Published:** 2013-09-25

**Authors:** M Bansal, M Khatri, V Taneja

**Affiliations:** * Department of Periodontics, Institute of Dental Studies and Technologies, Kadrabad, Modinagar Up, India; ** RT Dental Clinic, East of Kailash, New Delhi, India

**Keywords:** Periodontitis, Pneumonia, bacteraemia, interleukin

## Abstract

Respiratory diseases are responsible for a significant number of deaths and considerable suffering in humans. Accumulating evidence suggests that oral disorders, particularly periodontal disease, may influence the course of respiratory infections like bacterial pneumonia and chronic obstructive pulmonary disease (COPD). Oral periodontopathic bacteria can be aspirated into the lung causing aspiration pneumonia. The teeth may also serve as a reservoir for respiratory pathogen colonization and subsequent nosocomial pneumonia. The overreaction of the inflammatory process that leads to the destruction of the connective tissue is present in both periodontal disease and emphysema. This overreaction may explain the association between periodontal disease and chronic obstructive pulmonary disease. The mechanisms of infection could be the aspiration into the lung of oral pathogens capable of causing pneumonia, colonization of dental plaque by respiratory pathogens followed by aspiration, or facilitation of colonization of the upper airway by pulmonary pathogens by periodontal pathogens. The present article briefly reviews the epidemiologic evidence & role of periodontopathogens in causing respiratory infections.

## Introduction

Periodontal diseases are bacterial infections associated with bacteraemia, inflammation, and a strong immune response. Oral pathogens and inflammatory mediators such as interleukin-1 (IL-1) and tumour necrosis factor-α (TNF-α) from periodontal lesions immediately reach the blood stream inducing systemic inflammatory reactants such as acute phase proteins, and immune effectors including systemic antibodies to periodontal bacteria [**1**]. 

 Recent research has established that periodontal infection is a probable risk factor for cardiovascular disease, including atherosclerosis, myocardial infarction, stroke, diabetes, adverse pregnancy outcome & respiratory disorders. Recently, scientists and clinicians have begun to provide an increasing body of scientific evidence suggesting that moderate untreated periodontitis may affect an individual systemically, and may contribute to cardiovascular disease, diabetes and pre-term low birth weight [**2**]. Thus, the wheel has turned full circle. A new paradigm in dentistry in general, and periodontology in particular - Periodontal Medicine – has arrived. 

 Among these interactions is that between oral infections such as periodontitis and respiratory diseases. Respiratory diseases are responsible for significant morbidity and mortality in human populations. These diseases are widely prevalent and responsible for an extensive toll on human health and the cost of health care. Indeed, a recent report ranked lower respiratory infections as the third most common cause of mortality worldwide in 1990 (causing 4.3 million deaths), and chronic obstructive pulmonary disease as the sixth leading cause of mortality (2.2 million deaths). The anatomical continuity between the lungs and the oral cavity makes the latter a potential reservoir of respiratory pathogens. Yet an infective agent must defeat sophisticated immunological and mechanical defense mechanisms to reach the lower respiratory tract. The defense mechanisms are so efficient that, in healthy patients, the distal airway and lung parenchyma are sterile, despite the heavy bacterial load (106 aerobic bacteria and 107 anaerobic bacteria per milliliter) found in the upper airway [**3**]. An infection occurs when the host’s defenses are compromised, the pathogen is particularly virulent or the inoculum is overwhelming. The microorganisms may enter the lung by inhalation, but the most common route of infection is the aspiration of what pneumologists have long referred to as oropharyngeal secretions. Therefore, it is plausible that oral microorganisms might infect the respiratory tract. However, only recently has the role of the oral flora in the pathogenesis of respiratory infection been examined closely. 

 This paper will briefly describe the epidemiologic evidence and the mechanisms that support the role of oral bacteria in the process of respiratory infection [**4**]. 

### Bacterial pneumonia

 Pneumonia is an infection of the pulmonary parenchyma caused by a variety of infectious agents, including bacteria, mycoplasma, fungi, parasites, and viruses. The continuing emergence of antibiotic resistant bacteria (e.g., penicillin-resistant Pneumococci) suggests that bacterial pneumonia will assume an increasing importance in the coming years. 

 Pneumonia can be broadly classified into two types, with respect to their causative agents:

• Community acquired
• Hospital acquired (nosocomial)

Community acquired bacterial pneumonia is typically caused by pathogens that reside on the oropharyngeal mucosa such as Streptococcus pneumonia and Haemophilus influenza, Mycoplasma pneumonia, Chlamydia pneumonia, Legionella pneumophila, Candida albicans and anaerobic species. 

 Nosocomial pneumonia is often caused by bacteria that are not normally residents of the oropharynx but enter this milieu from the environment. These include Gram-negative bacilli (enteric such as Escherichia coli, Klebsiella pneumoniae, Serratia sps, Enterobacter sps.), Pseudomonas aeruginosa and Staphylococcus aureus [**5**]. 

### Chronic obstructive pulmonary disease (COPD) and Emphysema

 It is a condition characterized by chronic obstruction of airflow with excess production of sputum resulting from chronic bronchitis (CB) and/ or emphysema [**6**]. 

 Chronic bronchitis is the result of irritation of the bronchial airway, which causes an expansion of the proportion of mucus-secreting cells within the airway epithelium. 

 Emphysema is defined as the distention of the air spaces distal to the terminal bronchiole with destruction of the alveolar septa. 

### Risk factors for COPD:

 These include a history of prolonged cigarette smoking and genetic conditions such as the presence of a defective α1-antitrypsin gene, variant α1-antichymotrypsin, α2-macroglobulin, vitamin D binding protein, and blood group antigen genes. Other environmental risk factors include chronic exposure to toxic atmospheric pollutants (e.g., second hand smoke) [**7**]. 

### Pathogenesis of respiratory bacterial infection

 The lung is composed of numerous units formed by the progressive branching of the airways. The airway of each terminal respiratory unit (bronchiole, alveolar duct, alveolar sac and alveoli) is lined by epithelial cells in close proximity on their basal aspect to capillaries, which permits the efficient exchange of gases. The lower airways are normally sterile, despite the fact that the secretions of the upper airways are heavily contaminated with microorganisms seeded from the oral and nasal surfaces. Sterility of the lower airway is maintained by intact cough reflexes, the action of tracheobronchial secretions and mucociliary transport of inhaled microorganisms and particular material from the lower respiratory tract to the oropharynx, and immune and non immune defense factors (cell mediated immunity, humoral immunity, and polymorphonuclear leukocytes). The defense factors are contained within a secretion that coats the pulmonary epithelium. The secretion contains surfactant, other proteins such as fibronectin, complement and immunoglobulins. The lung also contains a rich system of resident phagocytic cells, which remove microorganisms and particulate debris [**8**]. 

 According to Scannapieco and Mylotte [**9**] microorganisms can contaminate the lower airways by four possible routes: 

 1) Aspiration of oropharyngeal contents, 

 2) Inhalation of infectious aerosols, 

 3) Spread of infection from contiguous sites, and 

 4) Haematogenous spread from extrapulmonary sites of infection (e.g., translocation from the gastrointestinal tract). 

### Role of oral bacteria in the pathogenesis of respiratory infection

 The oral bacterial species implicated in causing pneumonia and lung abscesses are Actinobacillus actinomycetemcomitans, Actinomyces israelii, Capnocytophaga species, Eikenella corrodens, Prevotella intermedia, Porphyromonas gingivalis and Streptococcus constellatus [**9**]. 

 Scannapieco [**10**] has proposed several mechanisms to explain the potential role of oral bacteria in the pathogenesis of respiratory infection:

• Aspiration of oral pathogens (such as Porphyromonas gingivalis, Actinobacillus actinomycetemcomitans, etc.) into the lung to cause infection,
• Periodontal disease-associated enzymes in saliva may modify mucosal surfaces to promote adhesion and colonization by respiratory pathogens, which are then aspirated into the lung,
• Periodontal disease-associated enzymes in saliva may destroy salivary pellicles on pathogenic bacteria to hinder their clearance from the mucosal surface
• Cytokines originating from periodontal tissues may alter respiratory epithelium to promote infection by respiratory pathogens.

### Aspiration of oral pathogens (**Fig. 1**)

**Fig. 1:**
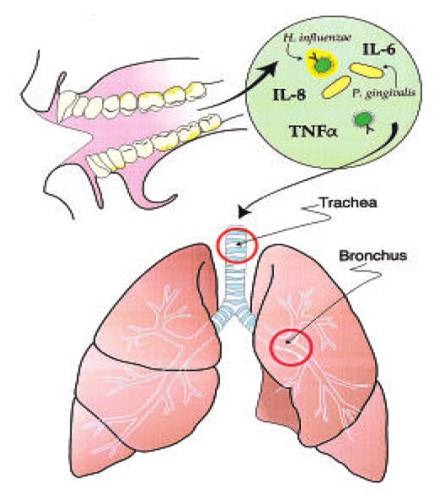
Aspiration of oral pathogens

Oral cavity is an important reservoir of bacterial pathogens that cause lung disease. Terpenning et al. [**11**] stated that the incidence of respiratory pathogen oropharyngeal colonization by respiratory pathogens appears to be more common in patients with teeth or dentures than in edentulous patients who do not wear dentures. Diminished salivation and salivary pH may promote colonization by respiratory pathogens; these conditions occur in ill patients and those receiving various medications.

Oral colonization by respiratory pathogens is common in institutionalized patients, especially those admitted to hospital ICUs and in the elderly who are debilitated or hospitalized [**11**].

#### Modification of mucosal surfaces by periodontal disease-associated enzymes in saliva

**Fig. 2:**
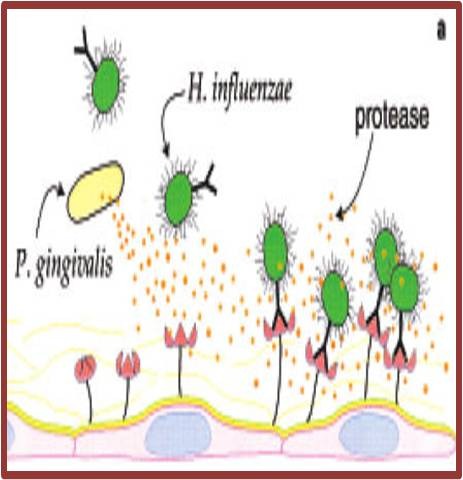
Pathogens produce enzymes that alter mucosal surface adhesion receptors for respiratory pathogens

 Woods et al. [**12**] reported that respiratory pathogens such as P. aeruginosa might adhere better to oral epithelial cells obtained from patients colonized by respiratory pathogens than to cells harvested from non-colonized patients. Trypsin treatment of epithelial cells from non-colonized patients in vitro resulted in increased adhesion of respiratory pathogens. This suggests that a mucosal alteration promoted enhanced bacterial adhesion of these bacteria. This alteration is perhaps the loss of fibronectin (by exposure to proteases) from the epithelial cell surface, which may unmask mucosal surface receptors for respiratory pathogen adhesins (**Fig. 2**).

#### Destruction of protective salivary pellicle by oral bacteria

**Fig. 3:**
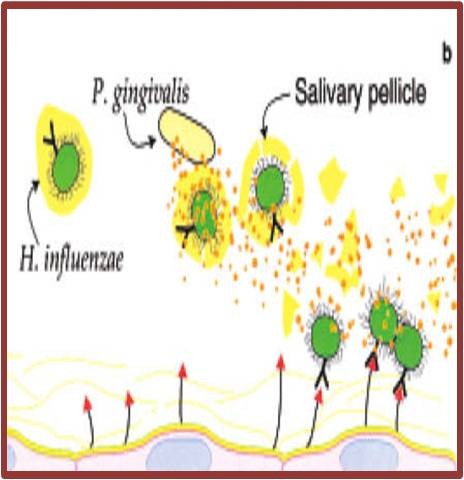
Enzymes degrade the pellicle on the oral pathogens, which prevent them from adhering to mucosal surface

**Fig. 4:**
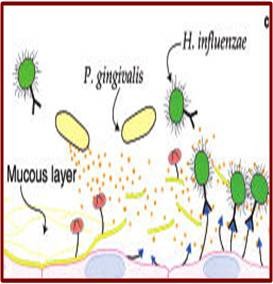
Enzymes degrade salivary pellicle on mucosal surface thereby exposing adhesion receptors for respiratory pathogens

 Subjects with poor oral hygiene may have elevated levels of hydrolytic enzymes (e.g. sialidase) in their saliva. These enzymes may process mucins to reduce their ability to bind to and clear pathogens such as H. influenza (**Fig. 3**). Conversely, the enzymes may process the respiratory epithelium to modulate the adhesion of such pathogens to the mucosal surface [**5**] (**Fig. 4**).

### Alterations in respiratory epithelium by cytokines

 Wilson et al. [**13**] stated that oral pathogens continuously stimulate the cells of the periodontium (epithelium cells, endothelial cells, fibroblasts, macrophages, and white blood cells) to release a wide variety of cytokines and other biologically active molecules. These are interleukin (IL)-1α, IL-1β, IL-6, IL-8, and TNF-α.

**Fig. 5:**
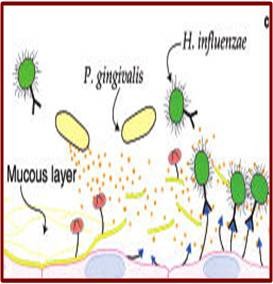
Cytokines upregulate the expression of adhesion receptors on the mucosal surfaces to promote respiratory pathogen colonization

 Oral bacteria in secretions encounter the respiratory epithelial surfaces and may adhere to the mucosal surface. These bound oral bacteria may stimulate cytokine production by mucosal epithelium. Cytokines originating from the oral tissues, which exit the gingival sulcus to be mixed with the whole saliva, may contaminate the distal respiratory epithelium to stimulate the respiratory epithelial cells. These stimulated cells may then release other cytokines that recruit inflammatory cells to the site. These inflammatory cells may release hydrolytic enzymes and other modifying molecules, resulting in damaged epithelium that may be more susceptible to colonization by respiratory pathogens [**9**] (**Fig. 5**).

Scannapieco & Ho [**14**] suggested that poor oral health might work in concert with other factors, such as continued smoking, environmental pollutants, viral infections, allergy and / or genetic factors which promote the progression and / or exacerbation of pulmonary disease. Subjects with the mean attachment loss (MAL) ≥ 3.0 mm had a higher risk of COPD than those having MAL < 3.0 mm. Lung function appeared to diminish with the increase in the periodontal attachment loss.

### Prevention of oral colonization by potential respiratory pathogens

 Because of the key role that the oropharyngeal bacterial colonization plays in the pathogenesis of bacterial pneumonia, several methods have been proposed to reduce or eliminate the colonization on susceptible patients such as those being treated by mechanical ventilation. Improved oral hygiene may decrease the occurrence of oropharyngeal colonization of PRPs and thus decrease the risk of respiratory disease. 

 Nord & Heindahl [**15**] suggested a method, called Selective Digestive Decontamination (SDD), which uses antibiotic topically applied to the surface of the gastrointestinal tract to reduce the carriage of pathogenic bacteria to prevent respiratory infection. The maintenance of good oral hygiene itself may reduce the oropharyngeal colonization by PRPs. However, chlorhexidine has a widespread use in dentistry to inhibit dental plaque formation, gingivitis and oral mucosal ulcerations, but it may not be effective against typical potential Gram-negative PRPs. This may be because chlorhexidine works best when teeth are free of plaque deposits, which is unlikely in ill patients admitted to a hospital.

## Conclusions

The above evidence suggests that improving oral hygiene might reduce the risk of respiratory infection among subjects who are at risk. A more rapid intervention would be the use of an oral disinfectant, but studies on the long-term use of such a medication are lacking. The treatment of periodontal diseases (either by repeated prescription of antibiotics or by clinical interventions) might be another way to reduce the incidence of respiratory infections. This literature review underlines the necessity for regular recalls among “at risk" patients and the introduction of specific oral hygiene courses for caregivers in long-term care institutions. Nonetheless, a causal association has not been proven, and more studies, in particular intervention studies, are needed. 
